# Metabolomic Comparison of Guava (*Psidium guajava* L.) Leaf Extracts Fermented by *Limosilactobacillus fermentum* and *Lactiplantibacillus plantarum* and Their Antioxidant and Antiglycation Activities

**DOI:** 10.3390/nu16060841

**Published:** 2024-03-14

**Authors:** Bo-Gyu Jun, Su-Hyun Kim, Seon-Hyeok Kim, Seong-Min Hong, Heaji Lee, Yunsook Lim, Sun-Yeou Kim, Choong-Hwan Lee

**Affiliations:** 1 Department of Bioscience and Biotechnology, Konkuk University, Seoul 05029, Republic of Korea; qhrb030@naver.com (B.-G.J.); kimsuhyun2019@naver.com (S.-H.K.); 2 College of Pharmacy, Gachon University, Incheon 21936, Republic of Korea; ssun0701@gachon.ac.kr (S.-H.K.); hongsm0517@gmail.com (S.-M.H.); sunnykim@gachon.ac.kr (S.-Y.K.); 3 Department of Food and Nutrition, Kyung Hee University, Seoul 02447, Republic of Korea; ji3743@khu.ac.kr (H.L.); ylim@khu.ac.kr (Y.L.); 4 Gachon Institute of Pharmaceutical Science, Gachon University, Incheon 21936, Republic of Korea; 5 Research Institute for Bioactive-Metabolome Network, Konkuk University, Seoul 05029, Republic of Korea

**Keywords:** probiotic fermentation, metabolomics, antioxidant activity, antiglycation activity

## Abstract

Probiotic fermentation of plant-based materials can lead to the generation of various bioactive substances via bacterial metabolites and the biotransformation of phenolic compounds. We compared the metabolic differences between fermentation by *Limosilactobacillus fermentum* KCTC15072BP (LFG) and fermentation by *Lactiplantibacillus plantarum* KGMB00831 (LPG) in guava leaf extract (0%, 0.5%, and 2% (*w*/*v*))-supplemented medium via non-targeted metabolite profiling. By performing multivariate statistical analysis and comparing the different guava leaf extract groups, 21 guava-derived and 30 bacterial metabolites were identified. The contents of guava-derived glucogallin, gallic acid, and sugar alcohols were significantly higher in LFG than they were in LPG. Similarly, significantly higher contents of guava-derived pyrogallol, vanillic acid, naringenin, phloretin, and aromatic amino acid catabolites were obtained with LPG than with LFG. LFG led to significantly higher antioxidant activities than LPG, while LPG led to significantly higher antiglycation activity than LFG. Interestingly, the fermentation-induced increase in the guava-leaf-extract-supplemented group was significantly higher than that in the control group. Thus, the increased bioactivity induced by guava fermentation with the Lactobacillaceae strain may be influenced by the synergistic effects between microbial metabolites and plant-derived compounds. Overall, examining the metabolic changes in plant-based food fermentation by differentiating the origin of metabolites provides a better understanding of food fermentation.

## 1. Introduction

Fermentation, recognized as one of the most ancient methods of food preparation worldwide, is a technology used to preserve foods by leveraging the growth and metabolic activities of microorganisms [1]. Lactic acid bacteria (LAB) are fermentative microorganisms that are widely utilized in the food industry and are known to produce metabolites that confer nutritional quality, sensory enhancement, and food preservation advantages [2]. LAB produce a variety of metabolites, such as organic acids, lactic acid, short-chain fatty acids (SCFA), bacteriocins, amino acids, and vitamins, during their metabolism [3]. LAB can also utilize their enzymes to biotransform the phenolics in plant substrates, such as flavonoids, phenolic acids, and tannins [4]. According to previous studies, LAB in plant-based foods increase their absorption rates by transforming plant-derived beneficial compounds [5,6,7]. LAB fermentation of plant-based foods generates diverse bioactive components, leading to increased bioactivities, such as antioxidant [8], anti-inflammatory [9], anti-obesity [10], and anti-diabetic [11,12] activities. LAB exhibit metabolic diversity according to the species and strain [13,14], and differences in their metabolic capabilities for plant material depending on the species and strain [15]. Accordingly, LAB exert various effects on the taste and quality of fermented foods [16]. The metabolism induced by LAB is affected by several factors, such as the presence of fermentable substrates or inhibitory factors, such as phenol compounds [17], highlighting the value of understanding the metabolic characteristics of the strains used in the food fermentation process and the metabolic capabilities of plant materials.

The guava tree (*Psidium guajava* L.), a member of the Myrtaceae family, is cultivated in tropical regions, such as India, Indonesia, Pakistan, Bangladesh, and South America [18]. Guava leaves (Psidii guajavae Folium), traditionally used to treat diseases, such as hypertension, cancer, inflammation, diabetes, and cough, are known to possess antioxidant, antitumor, and anticancer activities that are attributed to various phytochemicals, including phenolic compounds [19,20]. Various studies have reported increased antioxidant activity [21], anti-inflammatory effects [22], bioaccessibility, and anti-diabetic effects [23] of guava leaves fermented by microorganisms, particularly fungi. However, studies on guava leaves fermented by LAB with probiotic properties are scarce.

Reactive oxygen species (ROSs) are generated under oxidative stress and are the byproducts of aerobic metabolism. Increased ROS production results in harmful effects on important cellular structures, such as proteins, lipids, and nucleic acids [24]. Advanced glycation end products (AGEs) are stable end products formed from the reaction of the dicarbonyl compound methylglyoxal (MGO). AGEs increase ROS formation, which leads to diabetic complications [25]. Therefore, substances with antioxidant and antiglycation potential are garnering increased attention, and recent studies have reported enhanced antioxidant and antiglycation efficacy in fermented foods with probiotics [9,26,27].

In this study, a medium supplemented with guava leaf extract was fermented with *Limosilactobacillus fermentum* KCTC15072BP (LFG) and *Lactiplantibacillus plantarum* KGMB00831 (LPG), which are widely used in food fermentation. Through non-target analysis, metabolic changes were observed by comparing the metabolites produced by LAB. By examining the origin of the metabolites, we aimed to comprehensively interpret the metabolic differences attributed to the strains of the fermented substances, considering the metabolic characteristics of the bacteria and the transformation properties of plant-derived substances. By examining the antioxidant and antiglycation activities, we observed not only changes in activity due to fermentation, but also differences in activity between the two strains.

## 2. Materials and Methods

### 2.1. Chemicals and Reagents

HPLC-grade water, methanol, and acetonitrile were obtained from Thermo Fisher Scientific (Waltham, MA, USA). DeMan, Rogosa, and Sharpe (MRS) broth was obtained from DIFCO Laboratories, Inc. (BD Biosciences, Franklin Lakes, NJ, USA). Formic acid, reagent-grade methoxyamine hydrochloride, pyridine, N-methyl-N-(trimethylsilyl)-trifluoroacetamide (MSTFA), 6-hydroxy-2,5,7,8-tetramethylchroman-2-carboxylic acid (trolox), 1,1-diphenyl-2-picrylhydrazyl (DPPH), hydrochloride, 2,4,6-tris(2-pyridyl)-trizine (TPTZ), iron(III) chloride hexahydrate, sodium acetate, and acetic acid were obtained from Sigma-Aldrich (St. Louis, MO, USA).

### 2.2. Lactobacillus Strains and Culture Conditions

*Limosilactobacillus fermentum* KCTC (Korean Collection for Type Cultures) 15072BP and *Lactiplantibacillus plantarum* KGMB (Korean Gut Microbiome Bank) 00831 were maintained at −80 °C in MRS broth containing 20% glycerol (*v/v*). To activate the two strains of LAB, 0.1 mL of the strain stocks was cultured on MRS agar at 37 °C for 24 h in an anaerobic chamber. The cells were sub-cultured on MRS agar using the streaking method to obtain single colonies at 37 °C for 20 h. After the inoculation of single colonies into 5 mL of MRS broth in a 14 mL round-bottom tube (RB tube), the strains were sub-cultured at 37 °C for 18 h. The optical density (O.D) of the bacterial cultures was adjusted to 1.0 (approximately 2.02 × 10^9^ CFU/mL) via spectrophotometry at 600 nm. The activated LAB cells were used for further experiments. All bacterial cultures were carried out under anaerobic conditions in an anaerobic chamber (Coy Laboratory Products, Grass Lake, MI, USA) containing 10% CO_2_, 5% H_2_, and 85% N_2_.

### 2.3. Sample Preparation and Extraction

Three groups were established according to the culture substrate: not supplemented (0% group), supplemented with 0.5% (*w*/*v*) guava leaf extract (0.5% group), and supplemented with 2% (*w*/*v*) guava leaf extract (2% group). Each group was not fermented (NFG) or fermented with *Limosilactobacillus fermentum* (LFG) or *Lactiplantibacillus plantarum* (LPG) (Table 1). Three biological replicates were prepared for all samples.

Guava leaves were purchased in their dried form (Jeju Island, South Korea). Guava leaves for obtaining powder type were homogenized for 30 s by using an automill (TK-AM7-24, Tokken Inc., Chiba, Japan). Guava leaves powder (50 g) was suspended with a 500 mL of 50% ethanol in sterile Erlenmeyer flask (1 L) for 24 h at room temperature. The whole mixture is filtered with Whatman filter paper NO.2 (Whatman International, Maidstone, UK) and then subjected to a freeze-drying process to acquire the powdered extract. Guava leaf extracts were added to the MRS broth to obtain 0.5% (*w*/*v*) and 2% (*w*/*v*) concentrations for the bioconversion; MRS broth alone was used as a blank control. All culture media were sterilized at 121 °C for 15 min. The sterilized media were filtered through a 0.22 μm Polytetrafluoroethylene (PTFE) filter. Bacterial cultures adjusted to OD 1.0 were inoculated into non-supplemented medium and media supplemented with 0.5% or 2% (*w*/*v*) guava leaf extracts to obtain a final volume of 5% (*v/v*). The non-fermented medium and culture media were harvested at 24 h, and immediately centrifuged at 11,001 g for 10 min at 4 °C. To extract extracellular metabolites, the supernatant was collected and stored immediately under deep-freezing conditions (−80 °C). The supernatant of two strains was filtered through a 0.2 μm filter, with 100% methanol added in a 2:1 ratio. The samples were incubated at 200 rpm at 25 °C for 2 h on a rotary shaker, and then completely dried using a speed vacuum.

### 2.4. GC-TOF-MS Analysis

The dried extracts were re-dissolved to equal volumes using 50% MeOH and filtered using a 0.2 μm PTFE filter for GC-TOF-MS analysis. For derivatization, 100 μL of the re-dissolved sample was collected in 1.5 mL Eppendorf tubes and completely dried using a speed-vacuum concentrator. Derivatization involves oxidation and silylation. For oximation, 50 μL of methoxyamine hydrochloride (20 mg/mL in pyridine) was added to the dried extract, and the mixture was incubated for 90 min at 30 °C. Silylation was then performed by adding 50 μL of *N*-methyl-*N*-(trimethylsilyl) trifluoroacetamide to the mixture, and then the solution was incubated for 30 min at 37 °C. GC-TOF-MS analysis was performed using an Agilent 7890 B GC system with an Agilent 7693 autosampler and Pegasus BT TOF-MS (LECO, St. Joseph, MI, USA). Chromatographic separation was achieved using an Rtx-5MS column (30 m × 0.25 mm, 0.25 μm particle size; Restek Corp., St. Joseph, MI, USA), with helium as the carrier gas. The analytical methods and operational parameters were adapted from those described by Lee et al. [13]. Each derivatized sample was injected at 1 μL in a split of 15:1. All chromatographic runs were performed in random order to minimize systematic biases in the datasets.

### 2.5. UHPLC–Orbitrap–MS/MS Analysis

The dried extracts were re-dissolved to equal volumes using 50% MeOH and filtered using a 0.2 μm PTFE filter for UPLC–Orbitrap–MS/MS analysis. The UHPLC system was equipped with a Vanquish Binary Pump F (Thermo Fisher Scientific), coupled with an autosampler and a column compartment. Chromatographic separation was performed using a Phenomenex KINETEX^®^ C18 column (100 mm × 2.1 mm, 1.7 μm particle size; Torrance, CA, USA) and a mobile phase consisting of 0.1% (*v/v*) formic acid in water (solvent A) and 0.1% (*v/v*) formic acid in acetonitrile (solvent B). The following gradient condition was employed: 0–1 min, 5% B; 1–10 min, 5–100% B; 10–11 min, 100% B; 11–13 min, 100–5% B; and 13–15 min, 5% B. The flow rate, injection volume, and column temperature were 0.3 mL/min, 5 μL, and 40 °C, respectively. The mass spectra were recorded in the range 100–1500 *m*/*z* using an Orbitrap Exploris 120 Mass Spectrometer (Orbitrap MS, Thermo) coupled with a HESI-II(H-ESI) probe. The probe heater and capillary temperatures were set to 300 °C and 320 °C, respectively. The capillary voltage was set to 2.8 kV in the negative mode (positive mode, 3.5 kV). Five μL of each sample was injected in random orders.

### 2.6. Data Processing and Statistical Analysis

Three biological replicates were analyzed for each sample. Raw GC-TOF-MS data files were converted into NetCDF (*. cdf) format using LECO ChromaTOF software version 5.50.55.0. The UHPLC–Orbitrap–MS/MS raw data files were converted to NetCDF (*. cdf) format using Thermo Xcalibur software (version 2.1; Thermo Fisher Scientific). Retention time correction, peak detection, and alignment were performed using the XCMS online software version 3.7.1 (https://xcmsonline.scripps.edu, accessed on 2 November 2023) for LC-MS analysis and the Metalign software package version 1.0.0.1 (http://www.metalign.nl, accessed on 19 October 2023) for GC-MS analysis. Multivariate statistical analyses, including principal component analysis (PCA) and partial least squares discriminant analysis (PLS-DA), were performed using SIMCA-P+ software (version 15.0.2; Umetrics, Umea, Sweden). Significantly discriminant metabolites were selected based on their variable importance in projection (VIP > 1.0) values and one-way analysis of variance (ANOVA) based on *p* < 0.05. The metabolites were tentatively identified by comparing the mass fragment patterns, retention times, and MS analysis data to those of standard compounds under identical conditions or using available databases, including the National Institutes of Standards and Technology (NIST) Library (version 2.0, 2011; FairCom, Gaithersburg, MD, USA), PubChem (https://pubchem.ncbi.nlm.nih.gov/, accessed on 9 November 2023), and the Human Metabolome Database (HMDB; https://hmdb.ca/, accessed on 9 November 2023). PASW Statistics 18 (SPSS, Inc., Chicago, IL, USA) was used to determine significant differences (*p* < 0.05) based on one-way ANOVA and to calculate the correlation coefficient values for a correlation map. The correlation network map between metabolites with a Pearson’s correlation coefficient value higher than 0.5 and bioactivities was constructed using the Cytoscape software version 3.8.0 (https://www.cytoscape.org/, accessed on 30 January 2024).

### 2.7. Determination of the Antioxidant Activities and Antiglycation Activities

The antioxidant activity was measured using DPPH radical scavenging and ferric reducing antioxidant power (FRAP) assays. The DPPH and FRAP assays were performed as described by Lee et al. [28]. For the DPPH assay, 20 μL of the sample was mixed with 180 μL of the DPPH stock solution (0.2 mM in ethanol) in 96-well plates and allowed to react for 20 min at room temperature in the dark. Free radical absorbance by DPPH was measured at 515 nm. The FRAP assay was conducted using the FRAP reagent, which comprised 300 mM acetate buffer (pH 3.6), 20 mM iron (III) chloride, and 10 mM 2,4,6-tripyridyl-S-triazine (TPTZ) in 40 mM HCl (10:1:1, *v*/*v*/*v*). Briefly, 10 μL of the sample was mixed with 300 μL of FRAP reagent and incubated at room temperature for 6 min. The absorbance was measured at 570 nm. Antioxidant assays, such as the DPPH and FRAP assays, were conducted in triplicate, and the results are presented as the Trolox equivalent antioxidant capacity (TEAC) concentration (mM) per milligram of sample.

The AGE formation assay was performed as described by Kiho et al., with slight modifications [29]. Bovine serum albumin (BSA, 5 mg/mL) was mixed with MGO (10 mM) in phosphate-buffered saline (PBS, pH 7.4). Sodium azide (0.02%) was added to the reaction mixture and samples were incubated at 37 °C for 1 week. AGE formation was determined by measuring fluorescence at an excitation/emission wavelength of 355/460 nm using a VICTOR^TM^ X3 multilabel plate reader (Perkin Elmer, Waltham, MA, USA). Measurements were performed in triplicate, and the reaction mixture without MGO was used as the blank solution.

## 3. Results

### 3.1. Metabolic Profiling of the Guava Leaf Groups (0%, 0.5%, and 2%) Fermented by L. fermentum and L. plantarum

**Figure 1:**
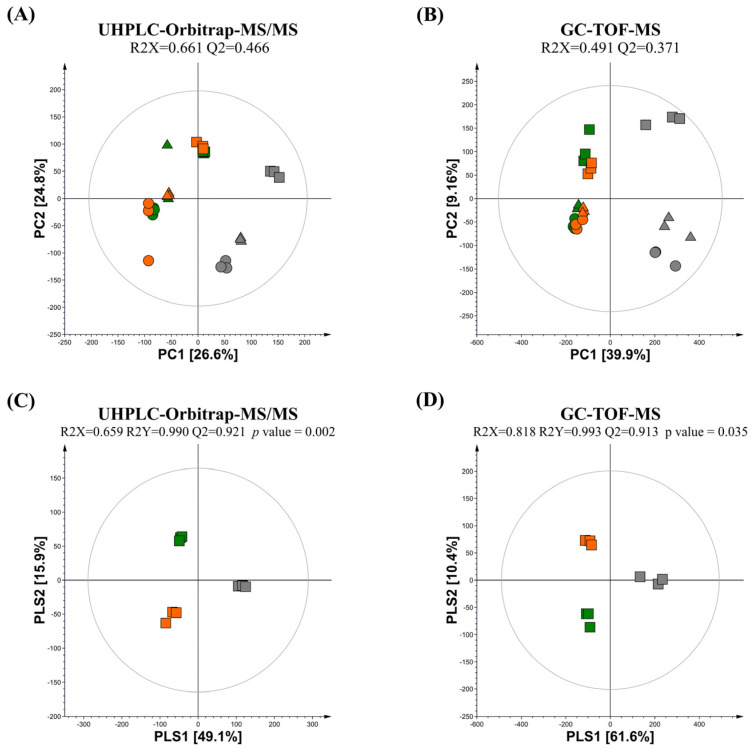
PCA and PLS-DA score plot of NFG, LFG, and LPG at (**A**,**B**) three (0%, 0.5%, and 2%) concentrations of the guava leaf extract and (**C**,**D**) 2% guava leaf extract. Multivariate analysis was conducted using datasets derived from (**A**,**C**) GC-TOF-MS and (**B**,**D**) UHPLC–Orbitrap–MS (NFG, non-fermentation (gray); LFG, guava leaves fermented by *L. fermentum* (green); LPG, guava leaves fermented by *L. plantarum* (orange); ●, 0% guava leaf extract group; ▲, 0.5% guava leaf extract group; ■, 2% guava leaf extract group).

Multivariate statistical analysis was conducted using the datasets obtained from UPLC–Orbitrap–MS/MS and GC-TOF-MS analyses for the guava leaf groups (0%, 0.5%, and 2%) not fermented (NFG) and fermented by *L. fermentum* (LFG) and *L. plantarum* (LPG). The principal component analysis (PCA) score plots obtained from UHPLC–Orbitrap–MS/MS and GC-TOF-MS revealed total variances of 51.4% (PC1, 26.6%; PC2, 24.8%) and 49.06% (PC1, 39.9%; PC2, 9.16%), respectively (Figure 1A,B). These PCA results clearly revealed differences in metabolomes between non-fermentation and LAB fermentation, and distinctions based on the concentration of the guava leaf extract.

**Figure 2:**
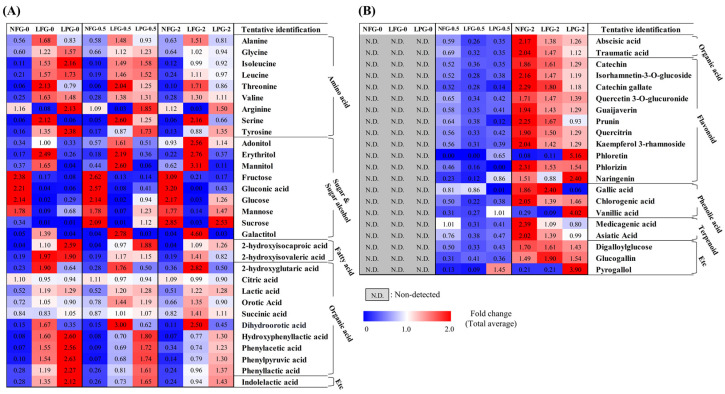
Heat map depicting the relative abundance of the (**A**) bacterial metabolites and (**B**) guava-derived metabolites of NFG, LFG, and LPG in the 0%, 0.5%, and 2% groups. Bacterial metabolites were selected based on detection in both non-supplemented medium and guava-supplemented medium. Guava-derived metabolites were only detected and selected from the group supplemented with the guava leaf extract (NFG, non-fermented; LFG, fermented by *L. fermentum*; LPG, fermented by *L. plantarum*).

Based on partial least squares discriminant analysis (PLS-DA) of the 2% guava leaf group, differences were found among the NFG, LFG, and LPG (Figure 1C,D). To examine the differences in bacterial metabolites and plant metabolite conversion between *L. fermentum* and *L. plantarum*, discriminant metabolites (VIP > 1.0, *p* value < 0.05) were screened based on the PLS-DA of the 2% group. A total of 51 discriminant metabolites, including 9 amino acids, 9 sugars and sugar alcohols, 12 organic acids, 2 fatty acids, 11 flavonoids, 3 phenolic acids, 2 terpenoids, 1 tannin, 1 benzene derivative, and 1 indole group derivative, were identified (Tables S1 and S2). Based on content comparison of the concentrations of the guava leaf extract for the entire group, 51 metabolites were distinguished based on their origins. The bacterial metabolites identified in the non-supplemented and supplemented guava leaf extract groups included 9 amino acids, 9 sugars and sugar alcohols, 2 fatty acids, 10 organic acids, and 1 indole group derivative (Figure 2A). Only 2 organic acids, 11 flavonoids, 3 phenolic acids, 2 terpenoids, 2 tannins, and 1 benzene were identified as guava-derived metabolites in the groups supplemented with the guava leaf extract (Figure 2B).

### 3.2. Metabolic Pathway Comparison of L. fermentum and L. plantarum Fermentation in the Guava Leaf Extract Groups (0%, 0.5%, and 2%)

**Figure 3:**
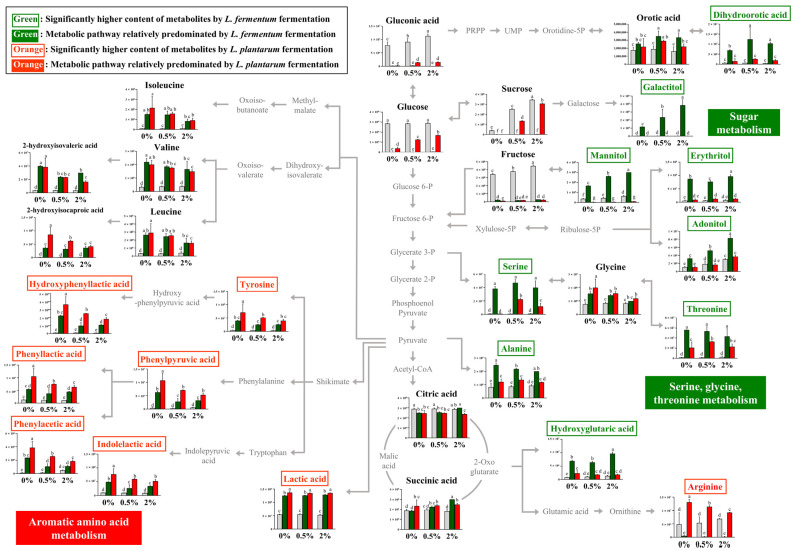
Schematic of the metabolic pathway and relative levels of NFG, LFG, and LPG metabolites detected in the different guava leaf extract groups (0%, 0.5%, and 2%). The metabolite pathways were adopted from the KEGG database and modified. The Y-axis of the graph represents peak areas of respective metabolites. Different letters indicate significant difference based on Duncan’s multiple-range test. Metabolites in green and orange font have relatively higher abundance when fermented by *L. fermentum* and *L. plantarum*, respectively (0%: 0% guava leaf group; 0.5%: 0.5% guava leaf group; 2%: 2% guava leaf group; 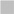: non-fermented (NFG); 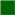: fermented by *L. fermentum* (LFG); 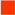: fermented by *L. plantarum* (LPG)).

The relative contents of bacterial metabolites (Figure 2A) in the 0%, 0.5%, and 2% groups after non-fermentation and LAB fermentation are depicted in the metabolic pathway (Figure 3). These contents were determined based on the peak area of each chromatogram. Metabolites showing strain-specific differences among the 0%, 0.5%, and 2% groups were analyzed.

By comparing the metabolism of *L. fermentum* and *L. plantarum* in the 0% group, metabolic differences attributable to *L. fermentum* and *L. plantarum* could be observed. Furthermore, by comparing the fermentation induced by the two strains in the 0%, 0.5%, and 2% groups, we could examine the metabolic effect of supplementing the guava leaf extract.

During sugar metabolism, sucrose, glucose, fructose, glyceric acid, and gluconic acid were reduced by fermentation with both bacteria compared with no fermentation. The sugar alcohol (galactitol, mannitol, erythritol, and adonitol) content was significantly higher with LFG, indicating a difference from that with LPG. During the metabolism of serine, glycine, and threonine, the relative contents of serine and threonine were markedly high with LFG. In the 0% group, serine production was observed as a unique feature of *L. fermentum* through fermentation, while no such effect was observed with *L. plantarum*. In addition, the levels of dihydroorotic acid, alanine, and hydroxyglutaric acid were significantly higher with LFG than LPG, exhibiting distinctive features. In contrast, the contents of tyrosine and aromatic amino acid catabolites, such as hydroxyphenyllactic acid, phenylpyruvic acid, phenyllactic acid, phenylacetic acid, and indoleacetic acid, were significantly higher with LPG than LFG, indicating a characteristic feature of aromatic amino acid metabolism. Lactic acid and arginine exhibited distinctively higher levels with LPG than LFG.

### 3.3. Bioconversion of the Guava-Derived Metabolites Fermented by L. fermentum and L. plantarum

**Figure 4:**
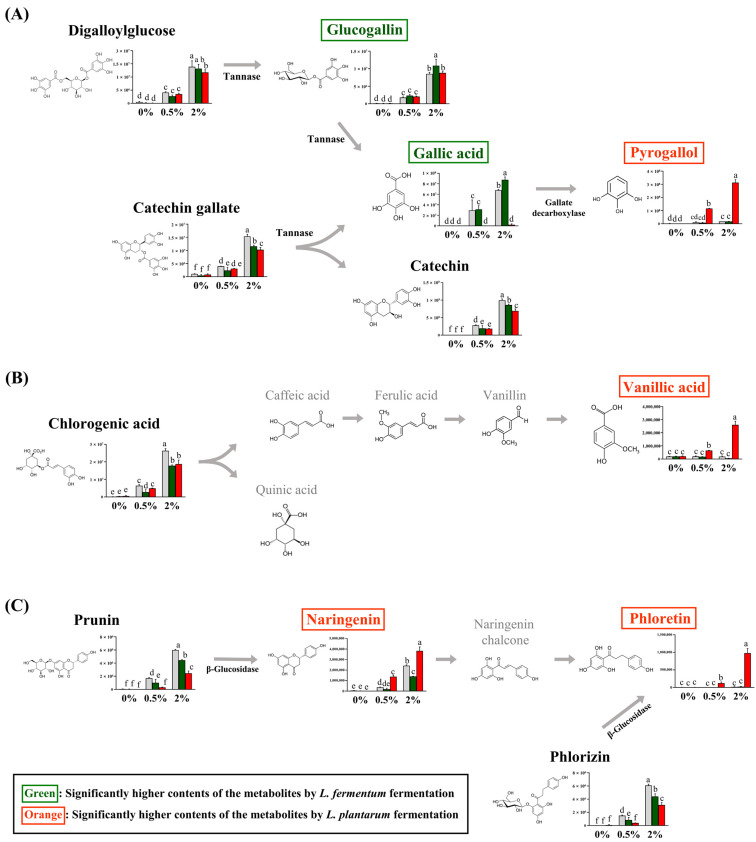
Proposed bioconversion pathways of (**A**) digalloyl glucose and catechin gallate, (**B**) chlorogenic acid, and (**C**) prunin and phlorizin. The selected compounds among the guava-derived metabolites are compared the their relative contents in NFG, LFG, and LPG in the groups supplemented with 0%, 0.5%, and 2% of the guava leaf extract. The Y-axis of the graph represents peak areas of respective metabolites. Black text indicates a decrease in metabolites by fermentation with *L. fermentum* and *L. plantarum*. Green text indicates an increase in metabolite with LFG alone. Orange text indicates an increase in metabolites with LPG alone. Different letters indicate significant differences based on Duncan’s multiple-range test. (0%: 0% guava leaf group; 0.5%: 0.5% guava leaf group; 2%: 2% guava leaf group; 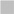: non-fermented (NFG); 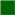: fermented by *L. fermentum* (LFG); 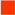: fermented by *L. plantarum* (LPG)).

Among the guava-derived metabolites (Figure 2B), we examined the bioconversion pathway of the metabolites increased by LAB fermentation (Figure 4), which was calculated based on the peak area of each chromatogram. Guava-derived metabolites showed different bioconversion patterns depending on the fermentation bacteria.

The contents of catechin gallate and digalloylglucose were significantly decreased with LFG and LPG compared to NFG. The glucogallin and gallic acid contents were significantly higher with LFG than those with LPG, while the pyrogallol content was significantly higher with LPG than LFG (Figure 4A). The chlorogenic acid content was reduced by LAB fermentation and was converted to vanillic acid with LPG (Figure 4B). Prunin was converted to naringenin, and phlorizin was converted to phloretin with LPG (Figure 4C). Although the levels of chlorogenic acid, prunin, naringenin, and phloretin decreased with LFG, no additional compounds exhibited an increase in our study.

### 3.4. Bioactivity in the Groups Supplemented with the Guava Leaf Extracts (0%, 0.5%, and 2%) and Fermented by L. fermentum and L. plantarum

**Figure 5:**
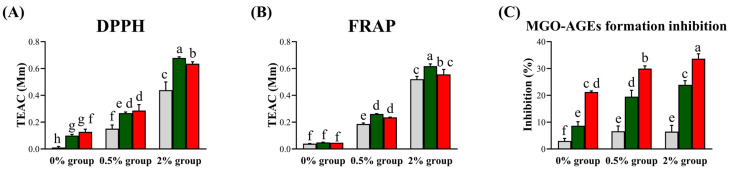
Bioactivities of NFG, LFG, and LPG in the groups supplemented with the guava leaf extract (0%, 0.5%, and 2%). Antioxidant activities based on the (**A**) DPPH assay and (**B**) Ferric reducing antioxidant power (FRAP) assay, and (**C**) antiglycation activity (MGO-AGEs formation inhibition assay). Values represent the averages of triplicate measurements (n = 3). The Y-axis of the (**A**) and (**B**) represents Trolox Equivalent Antioxidant Capacity, and in (**C**), it represents the degree of inhibition of MGO-AGEs formation. Each letter indicates significant differences according to Duncan’s multiple-range test (*p* < 0.05) (0% group: 0% guava leaf group; 0.5% group: 0.5% guava leaf group; 2% group: 2% guava leaf group; 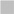: non-fermented (NFG); 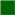: fermented by *L. fermentum* (LFG); 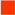: fermented by *L. plantarum* (LPG)).

To compare the bioactivities of LFG and LPG, we evaluated their antioxidant (DPPH and FRAP assays) and antiglycation (MGO-AGEs formation inhibition assay) activities (Figure 5). In all supplementation groups (0%, 0.5%, and 2%), increased activity due to LAB fermentation was observed, except for FRAP in the 0% group.

According to the antioxidant assay results, LFG led to a higher activity than LPG in the 2% group. Based on the DPPH results, LFG led to respective increases of 0.08, 0.11, and 0.24 TEAC compared to NFG, while LPG led to respective increases of 0.11, 0.13, and 0.20 TEAC compared to NFG in the 0%, 0.5%, and 2% guava leaf groups. No significant difference was observed for FRAP due to fermentation in the 0% group. However, in the 0.5% and 2% guava leaf groups, LFG induced increases of 0.08 and 0.09 TEAC, respectively, compared to NFG, while LPG induced increases of 0.05 and 0.03 TEAC, respectively, compared to NFG. Antiglycation activity was higher with LPG than LFG. In the 0%, 0.5%, and 2% guava leaf groups, LFG induced increases of 5.68%, 12.91%, and 17.43% in MGO-AGEs formation inhibition compared to NFG, whereas LPG induced increases of 18.27%, 23.36%, and 27.18% in MGO-AGEs formation inhibition compared to NFG.

**Figure 6:**
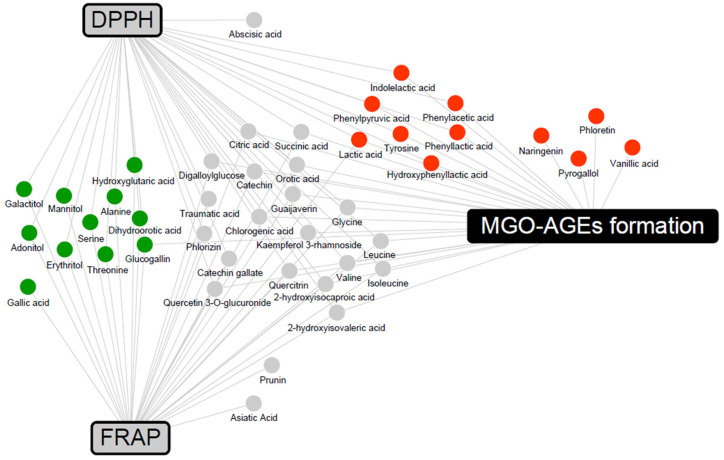
Correlation networks between the metabolites and bioactivity assays (DPPH, FRAP, and MGO-AGEs formation assay) in the 2% guava leaf group. The metabolites were selected based on a Pearson’s correlation coefficient value higher than 0.5. The box and circle symbols indicate bioactivities and the metabolites, respectively (gray color, antioxidant activity using DPPH and FRAP; black color, antiglycation activity, MGO-AGEs formation inhibition assay; 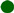, LFG-specific compounds; 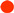, LPG-specific compounds; 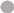, non-specific compound).

To determine the metabolites that may contribute to bioactivity, a correlation analysis between metabolites and bioactivity in the 2% group was conducted (Supplementary Figure S1). Metabolites with Pearson’s correlation coefficients higher than 0.5 were included in a network map (Figure 6). The antioxidant activities were associated with LFG-specific metabolites, whereas the antiglycation activities were associated with LPG-specific metabolites. In addition, flavonoids, branched-chain amino acids (valine, leucine, and isoleucine), and their catabolites (2-hydroxyisocaproic acid and 2-hydroxyisovaleric acid) were commonly associated with both antioxidant and antiglycation activities.

## 4. Discussion

In plant-based food fermentation, metabolites produced by microbial metabolism and those generated via the conversion of plant materials offer various benefits. To examine the metabolic changes occurring during plant additive fermentation using LAB, we established groups that were supplement with and without guava leaf extract (0% group and 0.5% and 2% groups, respectively) to distinguish between bacterial metabolites and guava-derived metabolites.

Although *L. fermentum* and *L. plantarum* belong to the same Lactobacillaceae family, differences in their metabolism during fermentation are evident (Figure 3). Significantly higher sugar alcohol content was observed with LFG than with LPG. The difference in sugar alcohol content during sugar metabolism was attributed to variations in the carbohydrate fermentation capabilities between the two strains. Both *L. fermentum* and *L. plantarum* have been reported to produce the sugar alcohol polyol [30]. However, the carbohydrates utilized during fermentation, including sugar alcohols, are found to differ. In a previous study, *L. fermentum* ATCC 14931 could not utilize mannitol whereas *L. plantarum* ATCC 149170 could ferment mannitol [31]. Even within the same species, differences in carbohydrate fermentation can be observed depending on the strain [32]. Therefore, the variation in sugar alcohol content may be attributed to strain-specific characteristics. In this experiment, the sugar alcohol content with LFG increased depending on the concentration of the guava leaf extract, which might be due to the increase in the content of carbohydrates (e.g., sucrose and fructose) resulting from the addition of the guava leaf extract. The differences in metabolite levels during serine, glycine, and threonine metabolism between *L. fermentum* and *L. plantarum* were likely due to genetic variations and differences in metabolic capabilities. *L. fermentum* can synthesize serine as it has two essential genes for serine synthesis from 3-phospho-D-glycerate [33]. In addition, *L. plantarum* tends to produce serine only under specific conditions, such as glucose stress [34]. Similarly, in the present study, an increase in serine levels in *L. plantarum* was observed only with the addition of guava leaf extract. Aminotransferases are involved in phenylpyruvic acid synthesis, and lactate dehydrogenase (LDH) is responsible for the production of phenyllactic acid, hydroxyphenyllactic acid, and indolelactic acid [35]. In this experiment, both LFG and LPG led to relatively higher levels of aromatic amino acid catabolites after fermentation than after NFG, with significantly higher levels observed with LPG than with LFG. In previous studies, the levels of phenyllactic acid and hydroxyphenyllactic acid in *L. plantarum* were higher than those in other LAB, including *L. fermentum*, which is consistent with the trend observed in this study [36]. Furthermore, LDH activity with phenylpyruvic acid was previously found to be highly correlated (R2 = 0.9606) with phenyllactic acid production [37]. Therefore, the difference between these two strains may be due to variations in the precursor levels of tyrosine, phenylalanine, and tryptophan; however, this difference could also be attributed to differences in enzyme activity between the two strains.

The conversion of guava-derived substances, observed only in the groups supplemented with the guava leaf extract, varied depending on the fermentation bacteria, suggesting that this result was likely due to differences in enzymes between *L. fermentum* and *L. plantarum*. Tannase, which converts digalloylglucose and catechin gallate to glucogallin and gallic acid, is present in LAB [38]. However, in the present study, an increase in the contents of glucogallin and gallic acid was only observed with LFG. The production of gallic acid through *L. fermentum* fermentation may exhibit different trends depending on the tannin content of the raw material and the strain [39]. In this study, such production was observed in the 2% group. The gallic acid content with LPG is relatively low, which might be due to the additional transformation of gallic acid to pyrogallol mediated by gallate decarboxylase [40]. Although gallate decarboxylase has not been found in *L. fermentum*, additional confirmation can be obtained through further transcriptome analysis. The chlorogenic acid content with LFG and LPG was lower than that with NFG. In addition, LPG resulted in higher concentrations of vanillic acid. This phenomenon was also observed in soybean hulls fermented with *L. plantarum* [41]. Although the chlorogenic acid content decreased with LFG, no additional compounds exhibiting an increase were observed in our study. The naringenin and phloretin contents produced by fermentation were higher with LPG than with NFG. The conversions of prunin to naringenin and phlorizin to phloretin have been reported to involve the enzyme, β-glucosidase [42,43]. Several studies have revealed the conversion of plant materials by beta-glucosidase in LAB [44]; however, in this experiment, the beta-glucosidase conversion patterns of the two strains were found to differ. Similarly, when flavanone metabolism was compared among the five LAB strains, all five strains exhibited beta-glucosidase activity; however, the converted metabolites exhibited significant differences depending on the strain [45]. This result suggests that differences in metabolism may occur through the action of enzymes other than LAB beta-glucosidase. Moreover, the differences in enzyme activity and substrate specificity between the two strains could explain this phenomenon [46]. Therefore, additional interpretation of the results of this experiment may be possible through transcriptome analysis of the two strains and further comparison of beta-glucosidase activity and affinity comparison of the substrate.

In this study, antioxidant activity (DPPH and FRAP) was significantly higher with *L. fermentum* fermentation, whereas antiglycation activity (MGO-AGEs formation inhibition) was significantly higher with *L. plantarum* fermentation. Furthermore, activity was higher with guava leaf extract supplementation compared to that without the guava leaf extract, indicating a synergistic effect of plant supplementation on LAB fermentation.

In the correlation network between metabolites and bioactivities (Figure 6), flavonoids, branched-chain amino acids, and their catabolites were associated with both antioxidant and antiglycation activities. Flavonoids are well-known antioxidants that exhibit antioxidant activity through various mechanisms, including scavenging of ROS [47]. According to a previous study, branched-chain amino acids have DPPH radical scavenging activity and upregulate the gene expression of the antioxidant protective catalase and glutathione peroxidase in vitro [48]. LFG-specific metabolites form a network associated with antioxidant activities and are known to possess antioxidant activity. Gallic acid stimulates various enzymatic and non-enzymatic antioxidant defenses, thereby preventing diet-induced oxidative stress and exhibiting activity against metabolic disorders, such as obesity, diabetes mellitus, and hyperlipidemia [49]. Previous studies have reported the antioxidant and hepatoprotective effects of glucogallin in vivo and in vitro [50]. The antioxidant activity of sugar alcohols has not been extensively reported. Among them, mannitol has been demonstrated to act as a cryoprotectant and free radical scavenger, influencing the activities of antioxidant enzymes, such as SOD, CAT, glutathione reductase (GR), peroxidase (POX), and ascorbate peroxidase (APX) [51]. In contrast, LPG-specific metabolites form a network associated with antiglycation activities; antiglycation-related activities have been reported. Guava-derived pyrogallol, vanillic acid, naringenin, and phloretin, and the aromatic amino acid catabolites (phenylpyruvic acid, hydroxyphenyllactic acid, phenyllactic acid, phenylacetic acid, and indolelactic acid) specific to *L. plantarum* were positively correlated with antiglycation activity (inhibition of MGO-AGEs formation). In previous studies, pyrogallol exhibited higher scavenging activity than other simple phenols (gallic acid, pyrocatechol, resorcinol, and phloroglucinol), trapping almost 90% of MGO [52]. Vanillic acid has been reported to significantly reduce glucose-induced BSA glycation and decrease AGE formation in immunohistochemical assays of the kidneys, indicating its antiglycation efficacy [53]. In addition, phloretin effectively inhibits the formation of AGEs by scavenging MGO in human umbilical endothelial cells [54]. Naringenin has been demonstrated to significantly inhibit AGE formation in bread and significantly reduce AGE-induced ROS production in RAW264.7 cells, confirming its antiglycation efficacy [55]. In previous studies, among the aromatic amino acids, phenyllactic acid and hydroxyphenyllactic acid were found to decrease ROS production in the mitochondria and neutrophils [56]. Additional explanations regarding the synergistic effect can be obtained by measuring the activity values of significantly derived bacterial and guava-derived metabolites.

## 5. Conclusions

Overall, we examined the metabolic changes induced by fermentation with *L. fermentum* and *L. plantarum* in media supplemented with guava leaf extract using non-targeted metabolic profiling and identified the origin of the metabolites. Although *L. fermentum* and *L. plantarum* belong to the same Lactobacillaceae family, clear differences in their metabolic characteristics and ability to convert plant substances were evident between the two strains based on pathway analysis. Further studies using multi-omics approaches are required to fully understand the differences in the metabolic characteristics and plant-derived material conversion of the bacterial strains used in this study. LFG-specific metabolites exhibited a greater association with antioxidant activity, whereas LPG-specific metabolites exhibited a stronger association with antiglycation activity, indicating differences in activity between the two strains. In addition, the changes in activity due to fermentation with the guava leaf extract supplement were more significant than those due to fermentation without this supplement, indicating a synergistic effect between probiotic fermentation and plant substances. This study, which sought to examine the metabolic characteristics of strains and conversion ability of plant materials and identify the origin of metabolites, could contribute to the discovery of bioactive substances that contribute to synergistic effects in future plant and microorganism co-fermentation processes. In addition, our approach could be useful for selecting microbial strains that can be used as starter cultures for food fermentation.

## Figures and Tables

**Table 1:** Information regarding the groups supplemented with the guava extract and the LAB strains used for fermentation.

Substrate	Inoculation	Abb ^a^	Symbol
MRS	-	NFG-0	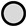
(0% group)	*Limosilactobacillus fermentum* KCTC15072BP	LFG-0	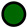
	*Lactiplantibacillus plantarum* KGMB00831	LPG-0	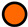
MRS with 0.5% (*w*/*v*) guava leaf extract	-	NFG-0.5	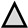
(0.5% group)	*Limosilactobacillus fermentum* KCTC15072BP	LFG-0.5	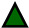
	*Lactiplantibacillus plantarum* KGMB00831	LPG-0.5	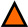
MRS with 2% (*w*/*v*) guava leaf extract	-	NFG-2	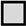
(2% group)	*Limosilactobacillus fermentum* KCTC15072BP	LPG-2	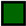
	*Lactiplantibacillus plantarum* KGMB00831	LFG-2	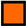

^a^ Abbreviation.

## Data Availability

Data is contained within the article.

## Supplementary Materials

The following supporting information can be downloaded from: https://www.mdpi.com/article/10.3390/nu16060841/s1, Table S1: List of significantly distinct metabolites from guava leaf extract supplemented medium by non-fermentation and LAB fermentation identified by UHPLC–Orbitrap–MS/MS; Table S2: List of significantly distinct metabolites from guava leaf extract supplemented medium by non-fermentation and LAB fermentation identified by GC-TOF-MS; Figure S1: Pearson’s correlation map between the relative abundance of discriminant metabolites and bioactivity assays (DPPH, FRAP, MGO-AGEs formation inhibition) of the 2% (*w*/*v*) guava-leaf-supplemented medium group. Each square indicates Pearson’s correlation coefficient values (r).
