# Supplementary material for: Metabolomic Comparison of Guava (Psidium guajava L.) Leaf Extracts Fermented by Limosilactobacillus fermentum and Lactiplantibacillus plantarum and Their Antioxidant and Antiglycation Activities
Source: Nutrients. 2024 Mar 14;16(6):841. doi: 10.3390/nu16060841 (PMC10974377; doi:10.3390/nu16060841)
Supplement: Supplementary file 1 [file nutrients-16-00841-s001.zip › nutrients-2901180-supplementary.pdf]

Table S1. List of significantly distinct metabolites from guava leaves extract supplemented medium by non-fermentation and LAB fermentation identified by UHPLC-Orbitrap-MS/MS.

No.	Tentativ identification	RT (min)[a]	VIP[b] 1	VIP 2	Measured mass (m/z)		Molecular formular	Fragment pattern	Δ ppm
					$[M-H]^-$	$[M+H]^+$			
	Amino Acids								
1	Arginine	0.61	0.21	1.77	173.1049	175.1187	C6H14N4O2	(-) 131 156 132 114	2.65
	Sugar & Sugar alcohols								
2	Galactitol	0.64	0.55	1.66	181.0719	205.0679[c]	C6H14O6	(-) 89 101 59 71 163 119	1.02
	Organic Acids								
3	Dihydroorotic acid	0.67	0.78	1.59	157.0257	-	C5H6N2O4	(-) 113 89 41 70 96	1.57
4	Phenylacetic acid	1.02	1.27	1.18	135.0454	-	C8H8O2	(-) 91	1.91
5	Hydroxyphenyllactic acid	1.02	1.35	1.10	181.0509	-	C9H10O4	(-) 163 135 119 71	1.5
6	Phenylpyruvic acid	1.02	1.33	1.11	163.0405	-	C9H8O3	(-) 119 135 128 120	2.45
7	Phenyllactic acid	5.06	1.38	1.06	165.0558	-	C9H10O3	(-) 147 119 72 103	0.79
8	Abscisic acid	6.76	1.37	0.97	263.1294	287.1254[c]	C15H20O4	(-) 153 219 204 163 111 125	1.95
9	Traumatic acid	7.52	1.36	1.07	227.1292	-	C12H20O4	(-) 183 165	1.18
	Flavonoids								
10	Catechin	1.18	1.23	1.17	289.0719	291.0858	C15H14O6	(-) 245 203 179 125 109	0.48
11	Isorhamnetin-3-O-glucoside	5.27	1.35	1.00	477.1044	501.0994	C22H22O12	(-) 313 169 163 433 386 300	1.09
12	quercetin 3-O-glucuronide	5.68	1.23	0.89	477.0671	479.0812	C21H18O13	(-) 301 178 151	-0.68
13	Catechin gallate	5.71	1.24	1.23	441.0828	443.0964	C22H18O10	(-) 169 189 245 125 411 205	0.24
14	Guaijaverin	5.92	1.40	1.00	433.078	435.0914	C20H18O11	(-) 300 301 178 151 271	0.64
15	Prunin	6.02	1.27	1.21	433.1144	435.1281	C21H22O10	(-) 271 151 272 300	0.83

16	Quercitrin	6.04	1.35	1.03	447.093	449.1071	C21H20O11	(-) 285 300 322 255	-0.6
17	Kaempferol arabinoside	6.19	1.36	0.97	417.0831	419.0971	C20H18O10	(-) 284 337 255 130	0.91
18	Phlorizin	6.21	1.32	1.10	435.1299	-	C21H24O10	(-) 273 167 125 179	0.49
19	Phloretin	7.26	0.85	1.54	273.0772	275.0911	C15H14O5	(-) 167 232 204 123	1.22
20	Naringenin	7.31	0.30	1.73	271.0615	273.0754	C15H12O5	(-) 151 119 177 93	1.21
	Phenolic acids								
21	Gallic acid	0.82	0.59	1.67	169.0145	171.0286	C7H6O5	(-) 125 81 97	1.66
22	Chlorogenic acid	1.13	1.33	1.01	353.088	355.1017	C16H18O9	(-) 191 173 135 61 233	0.59
23	Vanillic acid	6.4	0.84	1.55	167.0354	169.0493	C8H8O4	(-) 165 139 123 97 148	2.32
	Terpenoids								
24	Medicagenic acid	7.82	1.43	1.01	501.3225	503.3363	C30H46O6	(-) 455 339 357 227 274	0.63
25	Asiatic Acid	8.73	1.35	1.06	487.3434	489.3568	C30H48O5	(-) 387 322 342 363 302	1.06
	Etcs								
26	Glucogallin	0.81	1.04	0.64	331.0675	-	C13H16O10	(-) 271 169 211 241	1.31
27	Digalloylglucose	0.85	1.18	0.98	483.0782	-	C20H20O14	(-) 169 331 313 271 125 287 211	0.45
28	pyrogallol	0.91	0.86	1.55	125.0247	-	C6H6O3	(-) 81 97 107 78 69	2.05
29	Indolelactic acid	5.6	1.35	1.09	204.0668	206.0809	C11H11NO3	(-) 158 186 142 116 72	0.99

[a] RT, Retention time.

[b] VIP, Variable importance projection.

[c] Adduct ion is sodium, $[M+Na]^+$

Table S2. List of significantly distinct metabolites from guava leaves extract supplemented medium by non-fermentation and LAB fermentation identified by GC-TOF-MS.

No.	Tentative identification	RT(min)[a]	VIP[b] 1	VIP 2	Unique mass[c] *(m/z)*	Mass Fragment pattern	TMS[d]	ID
	Amino Acids							
1	Alanine	5.80	0.84	1.75	116	116, 117, 59, 118, 190, 100, 103, 218	2	STD
2	Valine	7.01	1.20	0.92	144	144, 218, 145, 100, 146, 219, 55, 128, 220	2	STD
3	Leucine	7.56	1.17	0.87	158	158, 159, 102, 100, 160, 232, 86, 260, 170, 233	2	STD
4	Isoleucine	7.77	1.12	0.80	158	158, 218, 159, 100, 160, 86, 232, 59, 69	2	STD
5	Glycine	7.91	1.21	0.97	174	174, 86, 175, 248, 100, 59, 176, 276, 133, 249	3	STD
6	Serine	8.42	0.77	1.14	204	204, 218, 205, 219, 103, 116, 206	3	STD
7	Threonine	8.65	0.78	1.28	219	117, 218, 219, 101, 57, 291, 100	3	STD
8	Tyrosine	12.62	1.18	1.19	179	179, 308, 180, 281, 309, 181, 293, 79, 355, 52	2	STD
	Sugar & Sugar alcohols							
9	Erythritol	9.70	0.71	1.93	217	217, 205, 103, 117, 204, 189, 191, 133	4	STD
10	Adonitol	11.40	0.78	1.85	217	217, 103, 205, 117, 129, 307, 218, 319	5	STD
11	Fructose	12.50	1.30	0.92	103	103, 217, 307, 133, 308, 218, 104	5	STD
12	Glucose	12.82	1.07	1.47	205	205, 319, 160, 103, 217, 320, 206	5	STD
13	Mannitol	12.95	0.47	2.11	205	103, 205, 217, 319, 117, 307, 133	6	STD
14	Gluconic acid	13.39	1.25	1.02	333	205, 103, 333, 217, 319, 292, 305	6	NIST
15	Sucrose	17.00	0.77	1.88	361	361, 217, 103, 362, 129, 169, 271, 363	8	NIST
17	Lactose	17.29	1.18	0.91	361	204, 217, 361, 205, 129, 103, 117, 169	8	STD
	Organic Acids							
16	Lactic acid	5.33	1.30	0.93	191	117, 117, 191, 66, 190	2	STD
17	Succinic acid	7.93	1.10	1.35	247	247, 55, 149, 56, 129	2	STD
18	Hydroxyglutaric acid	10.20	0.71	1.92	247	129, 247, 157, 203, 85, 149, 349	3	NIST
19	Orotic acid	11.53	0.89	1.39	254	254, 357, 255, 358, 256, 253, 269, 359, 174, 270	3	NIST

20	Citric acid	12.09	1.08	1.30	273	273, 274, 347, 375, 363, 211, 149	3	STD
	Fatty Acids							
21	2-hydroxyisovaleric acid	6.48	1.14	1.33	145	145, 219, 133, 146, 149, 55	2	STD
22	2-hydroxyisocaproic acid	7.19	1.28	0.95	159	159, 103, 69, 160, 133	2	STD

[a] RT, Retention time.

[b] VIP, Variable importance projection.

[c] m/z values are the selected ions for identification of derivatized metabolites

[d] TMS, Number of trimethylsilyl groups.

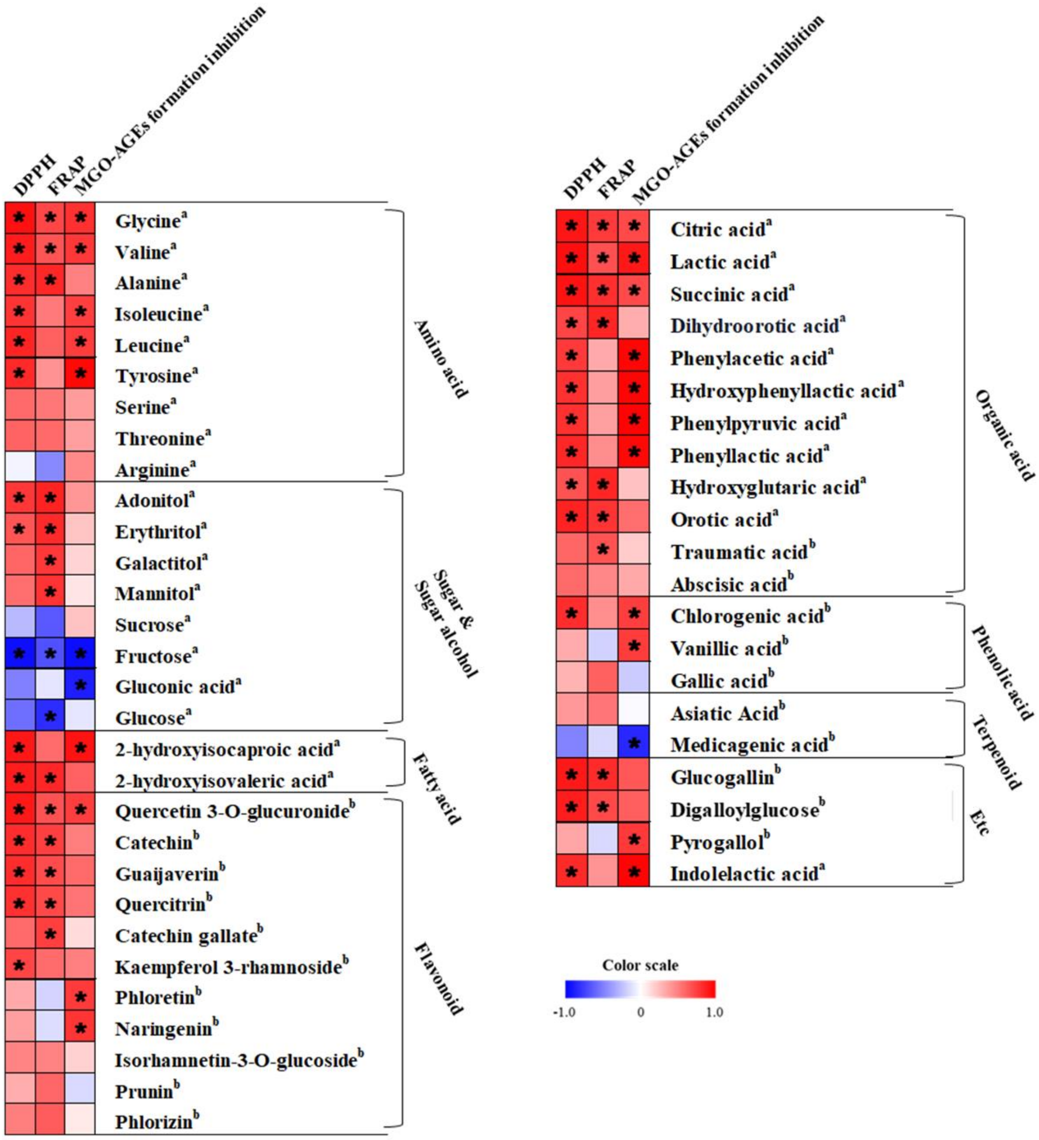

Figure S1. Pearson's correlation map between the relative abundance of discriminant metabolites and bioactivity assays (DPPH, FRAP, MGO-AGEs formation inhibition) of 2% (w/v) guava leaves supplemented medium group. Each square indicates Pearson's correlation coefficient values (r). Red and blue represent positive ($0 < r < 1$) and negative ($-1 < r < 0$) correlations, respectively. *: p-value <0.05; a: bacterial metabolites; b: guava-derived metabolites.
